# A dataset to model Levantine landcover and land-use change connected to climate change, the Arab Spring and COVID-19

**DOI:** 10.1016/j.dib.2024.110198

**Published:** 2024-02-19

**Authors:** Michael Kempf

**Affiliations:** Quaternary Geology, Department of Environmental Sciences, University of Basel, Bernoullistrasse 32, 4056 Basel, Switzerland

**Keywords:** Built-up change, Climate change, Migration, Land degradation, Drought, Jordan

## Abstract

The Levant is highly vulnerable to climate change and experiences prolonged heat waves that have led to societal crises and population displacement. In addition, the region has been impacted by further socio-political turmoil at least since 2010, including the Syrian civil war and currently the escalation of the so-called Israeli-Palestinian Conflict, which strained neighbouring countries like Jordan due to the influx of Syrian refugees and increases population vulnerability to governmental decision-making. Jordan, in particular, has seen rapid population growth and significant changes in land-use and infrastructure, leading to over-exploitation of the landscape through irrigation and unregulated construction activity. This article uses climate data, satellite imagery, and land cover information in a multicomponent trend analysis to illustrate the substantial increase in construction activity and to highlight the intricate relationship between climate change predictions and current socio-political development in the Levant. The analyses were performed using annual and seasonal composites of MODIS (Moderate Resolution Imaging Spectroradiometer) NDVI (Normalized Difference Vegetation Index) datasets with a spatial resolution of 250 m compared to climate indices of the GLDAS (Global Land Data Assimilation System) Noah Land Surface Model L4 dataset for the period 2001-2023. Surface reflectance and climatic parameters were then evaluated on the basis of socio-cultural factors, such as population dynamics, governmental decision-making, water withdrawal regulations, and built-up change as a result of large-scale migration processes. All analyses were conducted using R-software and can be reproduced and replicated using the code and the data provided in this article and the repository.

Specifications Table

**Table:** 

Subject	Agricultural Economics Mathematical Modelling Geographical Information System Real Estate Economics Climatology Global and Planetary Change Management, Monitoring, Policy and Law Hydrology and Water quality Water Science and Technology Political Science
Specific subject area	The Levant faces climate threats, socio-political instability, and over-exploitation of land, notably in Jordan, due to population growth and Syrian refugee influx since 2010. This article links climate data and built-up change, emphasizing the complex interplay with politics in the region.
Data format	Raw, Analyzed, Filtered, Derived
Type of data	Spatial data, raster data, vector data
Data collection	All statistical analyses and visualizations in this article were conducted using R software version 4.2.1, along with various R packages available from CRAN (Comprehensive R Archive Network), including terra, ncdf4, ggplot2, reshape2, gridExtra, gtools, ggbubr, gtable, and patchwork. The code and additional data related to this article are publicly accessible in the repository to this article. Climate parameters were analyzed using GLDAS Noah Land Surface Model L4 datasets, which cover the period from 1975-2014 (V2.0) and 2015-2023 (V2.1) at a global scale. These datasets, available through NASA's Earth Science Data Systems Program, consist of .nc4 files containing various climatic and environmental variables. Temperature, precipitation, specific humidity, canopy water evaporation, direct evaporation from bare soils, and evapotranspiration were extracted from these datasets and used to calculate annual mean values and sums. Trend analyses were performed using linear regression models highlighting slopes with >95% confidence as black dots. Mean raster values for each year across the study area are plotted in the code with a LOESS smoothing parameter of 0.3. Data from the Food and Agriculture Organization (FAO) was used to assess yield productivity trends from 1960 (1990) to 2021. The data was processed to estimate general trends and visualize them with the standard error of the estimated response. Additionally, built-up and land cover changes were analyzed using data from the European Commission and the Global Human Settlement Layer datasets (GHSL) for the period between 1975-2020 and 2022 at 100 m spatial resolution. Population development was plotted for urban, rural, and total estimates, utilizing data sources from FAO, the World Bank, and Worlddata.info.
	MODIS global NDVI (Normalized Difference Vegetation Index) datasets were obtained from the Earthdata server of the United States Geological Survey (USGS). These datasets covered the period from 2001-2022/3 and included seasonal composites (MAM, JJA, SON, DJF) and mean rasters. Trend analyses were conducted over 22 years, and mean raster statistics were computed for each year and plotted for each season, with high values indicating spectral greening and irrigated cropland and negative values indicating browning trends, built-up change, and infrastructural sprawl.
	*(continued on next page)*

**Table:** 

Data source location	GLDAS Noah Land Surface Model L4 datasets: NASA's Earth Science Data Systems (ESDS) Program, https://disc.gsfc.nasa.gov/datasets?keywords=GLDAS%20Noah%20Land%20Surface%20Model%20L4%20monthly&page=1 (last accessed 07th March 2023). Political boundaries were taken from https://www.geoboundaries.org (last accessed 7th of March 2023), and the naturalearthdata server, https://www.naturalearthdata.com/ (last accessed 5th of December 2023. FAO (Food and Agriculture Organisation of the United Nations) global data for yield productivity at annual resolution: https://www.fao.org/faostat/en/#data/QCL (last accessed 7th of March 2023). European Commission and the Global Human Settlement Layer datasets (GHSL): https://ghsl.jrc.ec.europa.eu/download.php?ds=bu (last accessed 7th of March 2023). Population development and water demand datasets derive from the FAO: https://www.fao.org/countryprofiles/index/en/?iso3=JOR (last accessed 4th of March 2023), the Worldbank: https://www.worldbank.org/en/home (last accessed: 04th of March 2023/ 13th of December 2023), Worlddata.info, e.g.: https://www.worlddata.info/asia/palestine/populationgrowth.php (last accessed 4th of March 2023). MODIS (Moderate Resolution Imaging Spectroradiometer) global NDVI (Normalized Difference Vegetation Index) datasets with a 16-day return period and a spatial resolution of 250 m: Earthdata server of the United States Geological Survey (USGS), MODIS/Terra Vegetation Indices 16-Day L3 Global 250m SIN Grid V006, https://lpdaac.usgs.gov/products/mod13q1v061/ (last accessed 7th of March 2023)
Data accessibility	Repository name: **zenodo** Data identification number: https://doi.org/10.5281/zenodo.10396148 Direct URL to data: https://zenodo.org/records/10396148
Related research article	Kempf, Michael (2024). Climate change, the Arab Spring, and COVID-19 - Impacts on landcover transformations in the Levant. Journal of Arid Environments, 221. https://doi.org/10.1016/j.jaridenv.2024.105132

## 1 Value of the Data

• This dataset offers statistically generated insights into the present socio-political patterns within a climate-vulnerable area (the Levant). It facilitates additional examinations and comprehension of the continuous population growth influenced by both climate and cultural factors, as well as the economic challenges being faced.
• Academics at the university level, as well as political stakeholders, non-governmental organizations (NGOs), and government bodies, have the opportunity to conduct re-analysis and derive insights from the trend analysis carried out using the provided code.
• Using the code or snippets of it for other analysis can easily facilitate the replication in other parts of the world and with different focus of underlying parameters.
• A focus of future studies can be put on drying-up processes of, but not only restricted to, the Levantine land surfaces, agricultural cropland and particularly climate-sensitive areas of semi-arid landscapes. Here, desertification and land degradation risks are especially high considering unregulated water extraction for crop cultivation, industry, or private consumption. Case studies with high resolution satellite imagery analysis (e.g., Sentinel-2 images with a 10 m spatial resolution and a frequent return period) can add local knowledge to large-scale trend analysis.
• A second potential application would be surface process modelling compared to archaeological site protection and heritage management in general. Erosion processes and land degradation as well as rapid built-up change lead to massive destruction of archaeological monuments in the very densely dispersed Levantine archaeological record. Because built-up change and surface transformations are accompanied by increased spheres of human activity, the risk of enhanced pollution by plastic waste or the uncontrolled spread of car tracks pose additional threat to the sensitive surface of the Near East. Probability maps of predicted surface vulnerability under climate change scenarios would help local authorities to inform about current ecological threats and potentially foster stronger interaction of the population with environmental change.
• As this repository includes both processed data and the code necessary to conduct the analysis, individuals can easily re-examine and reproduce the analyses even with little experience in the R programming language. By applying the code and globally accessible raw data from various sources to other climate indices and regions worldwide, this resource can support the replication of other findings on a global scale.
• This dataset relies entirely on openly accessible data and adheres to the FAIR principles of science. Employing these techniques in the instruction of geographical data analysis through the R programming language can promote a better understanding of the interactions between environmental and social sciences.

## 2 Data Description

In this section, the data structure and all code underlying the analysis is presented [1]. The code structure is explained first to make sure that the user does not get lost in the folders and sub-folders of the data [2]. Each code chunk refers to a single type of analysis, like extracting and reading data or performing trend analysis and plotting of results. Furthermore, the code uses different types of data, e.g., environmental proxy data, remote sensing data, and time series data. Each refer to a different directory to ensure a consistent data structure. Some of the code chunks can be written much easier and nested. However, to comment sufficiently and to facilitate reproducibility and replicability, the codes are provided separately with individual steps to better understand the process.

### 2.1 Code structure

**1_MODIS_NDVI_hdf_file_extraction.R**

This is the first code chunk that refers to the extraction of MODIS data from .hdf file format. The following packages must be installed and the raw data must be downloaded using a simple mass downloader, e.g., from google chrome. Packages: *terra*. Download MODIS data from after registration from: https://lpdaac.usgs.gov/products/mod13q1v061/ or https://search.earthdata.nasa.gov/search (MODIS/Terra Vegetation Indices 16-Day L3 Global 250m SIN Grid V061, last accessed, 09^th^ of October 2023). The code reads a list of files, extracts the NDVI, and saves each file to a single .tif-file with the indication “NDVI”. Because the study area is quite large, the user has to load three (spatially) different time series and merge them later. Note that the time series are temporally consistent.

**2_MERGE_MODIS_tiles.R**

In this code, the user loads and merges the three different stacks to produce large and consistent time series of NDVI imagery across the study area. Use the package *gtools* to load the files in 1, 2, 3, 4, 5, 6, etc. file order. Rasterstacks were merged one after the other. Eventually, single files were produced and named NDVI final_*consecutivenumber*.tif. Before saving the final output of single merged files, create a folder called “merged” and set the working directory to this folder, e.g., setwd(“your directory__MODIS/merged”).

**3_CROP_MODIS_merged_tiles.R**

Now the user wants to crop the derived MODIS tiles to our study area. Use a mask, which is provided as .shp file in the repository, named “MERGED_LEVANT.shp”. Then load the merged .tif files and crop the stack with the vector. Saving to individual files, and name them “NDVI_merged_clip_*consecutivenumber*.tif.

The repository provides the already clipped and merged NDVI datasets.

**4_TREND_analysis_NDVI.R**

Now, one canperform trend analysis from the derived data. The data is tricky as it contains 16-days return period across a year for the period of 22 years. Growing season sums contain MAM (March-May), JJA (June-August), and SON (September-November). December is represented as a single file, which means that the period DJF (December-February) is represented by 5 images instead of 6. For the last DJF period (December 2022), the data from January and February 2023 can be added. The code selects the respective images from the stack, depending on which period is under consideration. From these stacks, individual annually resolved growing season sums are generated and the slope is calculated. The user can then extract the p-values of the trend and characterize all values with high confidence level (0.05). Using the *ggplot2* package and the melt function from *reshape2* package, a plot of the reclassified NDVI trends together with a local smoother (LOESS) of value 0.3 can be created.

To increase comparability and understand the amplitude of the trends, z-scores were calculated and plotted, which show the deviation of the values from the mean. This has been done for the NDVI values as well as the GLDAS climate variables as a normalization technique.

**5_BUILT_UP_change_raster.R**

Now look at the landcover changes. This can be done using the *terra* package and raster data from here: https://ghsl.jrc.ec.europa.eu/download.php?ds=bu (last accessed 03. March 2023, 100 m resolution, global coverage). Here, one can download the temporal coverage that is aimed for and reclassify it using the code after cropping to the individual study area. Here, the raster data was summed up to characterize the built-up change in continuous values between 1975 and 2022.

**6_POPULATION_numbers_plot.R**

For this plot, one needs to load the .csv-file “Socio_cultural_political_development_database_FAO2023.csv” from the repository. The *ggplot* script produces the desired plot with all countries under consideration.

**7_YIELD_plot.R**

In this section, the user can plot the country productivity from the supplement in the repository “yield_productivity” (e.g., “Jordan_yield.csv”). Each of the single country yield datasets is plotted in a *ggplot* and combined using the *patchwork* package in R.

**8_GLDAS_read_extract_trend**

The last code provides the basis for the trend analysis of the climate variables used in the paper. The raw data can be accessed from this link https://disc.gsfc.nasa.gov/datasets?keywords=GLDAS%20Noah%20Land%20Surface%20Model%20L4%20monthly&page=1 (last accessed 9th of October 2023). The raw data comes in .nc file format and various variables can be extracted using the [“^a variable name”] command from the spatraster collection. Each time you run the code, this variable name must be adjusted to meet the requirements for the variables (see this link for abbreviations: https://disc.gsfc.nasa.gov/datasets/GLDAS_CLSM025_D_2.0/summary, last accessed 09th of October 2023; or the respective code chunk when reading a .nc file with the ncdf4 package in R) or run print(nc) from the code or use names(the spatraster collection).

Choosing one variable, the code uses the MERGED_LEVANT.shp mask from the repository to crop and mask the data to the outline of the study area.

From the processed data, trend analysis are conducted and z-scores were calculated following the code described above. However, annual trends require the frequency of the time series analysis to be set to value = 12. Regarding, e.g., rainfall, which is measured as annual sums and not means, the chunk r.sum=r.sum/12 has to be removed or set to r.sum=r.sum/1 to avoid calculating annual mean values (see other variables). Seasonal subset can be calculated as described in the code. Here, 3-month subsets were chosen for growing seasons, e.g. March-May (MAM), June-July (JJA), September-November (SON), and DJF (December-February, including Jan/Feb of the consecutive year).

From the data, mean values of 48 consecutive years are calculated and trend analysis are performed as describe above. In the same way, p-values are extracted and 95 % confidence level values are marked with dots on the raster plot. This analysis can be performed with a much longer time series, other variables, ad different spatial extent across the globe due to the availability of the GLDAS variables.

**9_workflow_diagramme** this simple code can be used to plot a workflow diagram and is detached from the actual analysis.

### 2.2 Folder structure

**Fig. 1:**
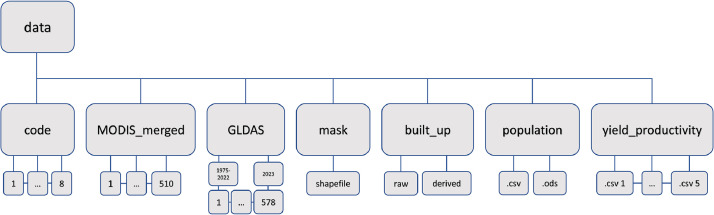
Data folder structure.

The main folder is named “data”, in which the following subfolders are stored (see Fig. 1):

“code” stores the above described 9 code chunks to read, extract, process, analyse, and visualize the data.

“MODIS_merged” contains the 16-days, 250 m resolution NDVI imagery merged from three tiles (h20v05, h21v05, h21v06) and cropped to the study area, n=510, covering January 2001 to December 2022 and including January and February 2023.

“mask” contains a single shapefile, which is the merged product of administrative boundaries, including Jordan, Lebanon, Israel, Syria, and Palestine (“MERGED_LEVANT.shp”).

“yield_productivity” contains .csv files of yield information for all countries listed above.

“population” contains two files with the same name but different format. The .csv file is for processing and plotting in R. The .ods file is for enhanced visualization of population dynamics in the Levant (Socio_cultural_political_development_database_FAO2023.ods).

“GLDAS” stores the raw data of the NASA Global Land Data Assimilation System datasets that can be read, extracted (variable name), and processed using code “8_GLDAS_read_extract_trend” from the respective folder. One folder contains data from 1975-2022 and a second the additional January and February 2023 data.

“built_up” contains the landcover and built-up change data from 1975 to 2022. This folder is subdivided into two subfolder which contain the raw data and the already processed data. “raw_data” contains the unprocessed datasets and “derived_data” stores the cropped built_up datasets at 5 year intervals, e.g., “Levant_built_up_1975.tif”.

## 3 Experimental Design, Materials and Methods

All data has been gathered from open source repositories and open science hubs (see data description for sources and processing). In this study, all statistical analyses and graphical representations were performed utilizing the R software version 4.2.1 (R Core Team, 2021), along with several packages from the Comprehensive R Archive Network (CRAN), including terra, ncdf4, ggplot2, reshape2, gridExtra, gtools, ggbubr, gtable, tidyterra, and patchwork. The entire code and supplementary data for this research are publicly accessible in the provided repository.

Climate data was extracted from the Global Land Data Assimilation System (GLDAS) Noah Land Surface Model L4 datasets, which offer monthly temporal resolution and a spatial resolution of 0.25 × 0.25 degrees [3,4]. These datasets span two periods, namely 1975-2014 (V2.0) and 2015-2022 (V2.1), and encompass a global scope. The data, available in .nc4 file format, contains various climatic and environmental variables that were separated into individual raster files. To restrict the global datasets to the geographical area of interest, which comprises Jordan, Palestine, Israel, Syria, and Lebanon, political boundary data was obtained from the geoboundaries.org source and the naturalearthdata server at https://www.naturalearthdata.com/ (last accessed 5th of December 2023).

From the stacked datasets, several climate variables can be extracted, such as temperature (Tair_f), precipitation (Rainf_f), specific humidity (Qair_f), canopy water evaporation (ECanop), direct evaporation from bare soils (ESoil), and Evapotranspiration (Evap). Annual mean values for the variables and sums for Rainf_f were calculated. Subsequently, using annual stacks, trend analyses were performed employing a linear regression model and the slope was computed and can be plotted using the code in the repository. The extracted p-values, and all slope values exceeding a 95% confidence threshold were marked as black dots in the plot. The next plot illustrates the mean raster values for each year across the study area, plotted using a LOESS smoothing parameter of 0.3, which is a non-parametric smoothing technique involving linear regression between x and y.

The FAO (Food and Agriculture Organisation of the United Nations) supplied global yield productivity data at an annual resolution. Country-specific data was extracted, and yield productivity was employed to estimate overall food productivity from 1960 (1990) to 2021. The data was categorized for trend estimation and displayed using the standard error of the estimated response (see the code provided in this article).

Built-up and landcover change were evaluated using data from the European Commission and the Global Human Settlement Layer datasets (GHSL). The datasets, which cover 5-year intervals between 1975 and 2020, and up to 2022 with a spatial resolution of 100 meters, were cropped to the study area. These datasets were converted into binary raster format (0=NA, 1=built-up) and aggregated to visualize positive built-up change.

Population changes in each country can be plotted for urban (Jordan, Syria, Israel), rural (Jordan, Syria, Israel), and total (Lebanon, Palestine, hereafter referred to as urban population) estimates. Data sources included the FAO (https://www.fao.org/countryprofiles/index/en/?iso3=JOR, last accessed on March 4, 2023), the Worldbank (https://www.worldbank.org/en/home, last accessed on March 4, 2023), and Worlddata.info (e.g., https://www.worlddata.info/asia/palestine/populationgrowth.php, last accessed on March 4, 2023).

MODIS (Moderate Resolution Imaging Spectroradiometer) global NDVI (Normalized Difference Vegetation Index) datasets with a 16-day temporal resolution and a spatial resolution of 250 meters were obtained from the United States Geological Survey (USGS) Earthdata server (MODIS/Terra Vegetation Indices 16-Day L3 Global 250m SIN Grid V006, https://lpdaac.usgs.gov/products/mod13q1v061/, last accessed on March 7, 2023) [5]. NDVI is a vegetation performance indicator that utilizes red and infrared spectral bands to measure photosynthetic activity and distinguish plant cover from other surfaces. Higher NDVI values indicate increased photosynthetic activity [6], [7], [8], [9].

The dataset spans from 2001 to 2022, with 23 images per year and additional images for January and February 2023, creating seasonal time series trends. Three temporal series were downloaded of the tiles MOD13Q_h21v05.006, MOD13Q1_h20v05.006, and MOD13Q1_h20v06.006 and the NDVI layers were extracted from the .hdf files. Seasonal composites (MAM, March-May; JJA, June-August; SON, September-November; DJF, December-February) and mean rasters were calculated. Trend analyses over 22 years were performed as described above and in the data description and the code [1,10]. Mean raster statistics were computed for each year and plotted for each season from 2001 to 2022, including January and February 2023 for DJF. Total trends can be visualized as raster images, including only pixels with a 95% confidence level. High values indicate spectral greening and irrigated cropland, while negative values suggest browning trends, built-up change, and infrastructural expansion [10].

## Limitations

Typical limitations of NDVI and remote sensing applications are the spatial and temporal resolution of the data available to now. Here, the regression model is based on 510 images covering 22 years, which can be a limiting factor for a trend analysis with regard to long-term trends not captured by the data. GLDAS provides global coverage, however, the resolution could obfuscate local and microregional ecological feedbacks, particularly in climate-sensitive areas of the world. Here, local high resolution case studies can help to re-evaluate landcover trends in climatically sensitive areas of the world and contribute to the large-scale modelled results. Currently (as of February 2024), the region covered in this article is at war, which is a limiting factor for analysis and field work *per se*. The situation, now politically and socially in a severe state for the local population, will enhance the pressure on governmental decision-making processes in the Near-East with a particular focus on the Israeli-Palestine relationship. Climate change induced trends could probably be massively reinforced by political actions in the future, which makes it more difficult to predict potential scenarios. Due to the short time period under consideration in the trend analysis, ecological collapse and rapid depletion of the local water resources can lead to cascading feedbacks that could not have been expected by the time series alone.

The presented data is, eventually, the basis for a model that describes empirically observed data. The multi-facetted “reality” is, however, controlled or impacted by dozens of external and internal parameters that cannot be captured in a simple trend analysis of climate variables alone. Again, a model does not describe real circumstances but rather potential and simplified characteristics of circumstances with regard to an empirically observed item. The model can show realistic patterns or trends but is never real. One should keep in mind that changing information depth, sample number, or case study region can lead to abandoning the theory presented in the accompanied research paper [1] and the formulation of another (adopted) model or theory.

## CRediT authorship contribution statement

**Michael Kempf:** Conceptualization, Methodology, Software, Validation, Formal analysis, Investigation, Resources, Data curation, Writing – original draft, Writing – review & editing, Visualization, Supervision, Project administration, Funding acquisition.

## Data Availability

A dataset to model Levantine landcover and land-use change connected to climate change, the Arab Spring and COVID-19 [Data set] (Original data) (Zenodo).
